# Correlation between thyroid fine needle aspiration and pathological examination: a 10 year retrospective study

**DOI:** 10.31744/einstein_journal/2023AO0418

**Published:** 2023-12-01

**Authors:** Rodrigo Ribeiro e Silva, Vinicius Ribas de Abreu Borges, Alexandre Grunfeld Starling Jardim, Maria Luisa Hostin Volpi, Leonora Zozula Blind Pope, Manuella Zattar Medeiros

**Affiliations:** 1 Department of Medicine Universidade da Região de Joinville Joinville SC Brazil Department of Medicine , Universidade da Região de Joinville , Joinville , SC , Brazil .; 2 Pathological Anatomy Laboratory Hospital Dona Helena Joinville SC Brazil Pathological Anatomy Laboratory , Hospital Dona Helena , Joinville , SC , Brazil .

**Keywords:** Thyroid neoplasms, Thyroid nodule, Biopsy, fine-needle, Thyroidectomy

## Abstract

**Objective:**

To establish the diagnostic performance of fine-needle aspiration in detecting benign and malignant neoplasm in comparison with post-thyroidectomy histopathological findings among patients who received a thyroidectomy.

**Methods:**

Retrospective observational data collected between 2011–2021 were included from patients who received partial or total thyroidectomy. The Bethesda system was used to classify neoplasms from fine-needle aspiration procedures as benign or malignant. Sample characteristics, diagnostic accuracy, sensitivity, specificity, and predictive values were evaluated.

**Results:**

Patients (n=360) who underwent thyroidectomy were analyzed, of whom 142 (39.4%) and 218 (60.6%) had benign and malignant neoplasms, respectively. Using the Bethesda system, 23 (6.4%) were classified as unsatisfactory result (BI), 83 (23.1%) as benign (BII), 50 (13.9%) as atypia of undetermined significance (BIII), 23 (6.4%) as suspected follicular or Hürthle cell neoplasia (BIV), 102 (28.3%) as suspected malignancy (BV) and 79 (21.9%) as malignant (BVI). The fine-needle aspiration diagnostic accuracy for carcinomas was 92%, while the sensitivity and specificity were 94.4% and 86.9%, respectively. The negative and positive predictive values were 87.9% and 93.9%, respectively.

**Conclusion:**

Fine-needle aspiration has high diagnostic accuracy, sensitivity and specificity, and is a reliable test for distinguishing between benign and malignant thyroid pathologies.

## Summary

In Brief

After analyzing 360 patients who underwent thyroidectomy, 60.6% of neoplasms were classified as malignant. Fine-needle aspiration demonstrated reliable diagnostic parameters for distinguishing between benign and malignant thyroid nodules. Compared to histopathology, fine-needle aspiration had a diagnostic accuracy rate of 92% as well as high sensitivity and specificity.

## Abstract (key points)

Highlights

Considering Bethesda V and VI as malignant findings, the fine-needle aspiration accuracy was 92%.

Fine-needle aspiration showed 94.4% sensitivity and 86.9% specificity compared to histopathology.

We found a positive and negative predictive value of 93.9%and 87.9%, respectively.

When Bethesda III and IV were included as malignant, accuracy reduced to 80.1%.

## Graphical abstract

**Figure:**
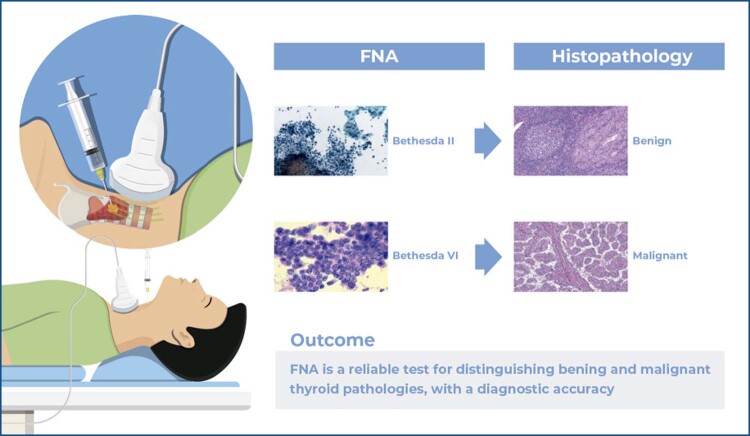

## INTRODUCTION

Thyroid nodules are common in clinical practice and can be caused by numerous diseases. ^( 1 )^ In geographic regions where iodine supplementation is effective, the incidence of palpable nodules is 5% and 1% in women and men, respectively. ^( 2 )^ However, with the most modern detection techniques, such as thyroid ultrasonography, incidence of thyroid nodules is 50% in the general population. ^( 2 , 3 )^

Approximately 95% of thyroid nodules are considered benign, of which adenomatous nodules, isolated Hashimoto’s thyroiditis, and cysts are the main types. ^( 1 )^ Early detection of malignant thyroid lesions is essential for better prognosis and management of thyroid neoplasms. ^( 3 , 4 )^ Without presence of distant metastases at the initial diagnosis, the 30-year mortality rate is approximately 6% to 10%. However, mortality rates can reach 65% in patients with stage IV carcinoma. ^( 4 )^

Thyroid carcinoma is the most common neoplasm of the endocrine system, accounting for 1-2% of all cases of cancer worldwide. ^( 4 )^ In 2013, the incidence rate of thyroid carcinoma was estimated at 24 and 12.9 cases per 100,000 people in Brazil and the U.S., respectively. ^( 1 , 5 )^ It is the fourth most common neoplasm in women, and recent increases in disease incidence are associated with improved sensitivity of diagnostic examinations and detection of small lesions. ^( 5 )^

Isolated clinical examinations of the thyroid cannot reliably differentiate benign from malignant nodules. ^( 1 )^ Although thyroid ultrasound is a low-cost, relatively accessible, and non-invasive test, the nodule features observed by ultrasound cannot accurately identify malignant lesions. ^( 2 )^ For this reason, fine-needle aspiration (FNA) is currently considered the gold standard diagnostic method for thyroid nodules. ^( 2 , 3 )^ The FNA test consists of a cytopathological analysis of morphological changes in isolated cells of the gland obtained via fine-needle puncture. ^( 5 )^

Detection of malignant tumors has increased since the introduction of the FNA method. ^( 3 )^ Furthermore, benign results help prevent unnecessary surgeries. ^( 3 , 4 )^ The use of the technique reduced the number of thyroidectomies by 50%. ^( 3 , 5 )^ Its advantages include minimal invasiveness and high sensitivity, precision, and specificity. ^( 3 )^ However, the test does not assess the degree of malignancy or invasiveness of the lesion as these parameters require more detailed histological analyses. ^( 5 )^

Considering the high prevalence of thyroid nodules in the population and the importance of early diagnosis and treatment of carcinomas, evaluating the accuracy, sensitivity, and specificity of FNA for detecting neoplasms is crucial. Improving these diagnostic procedures can have a substantial impact on patients and healthcare providers.

## OBJECTIVE

To establish the diagnostic performance of fine-needle aspiration in detecting benign and malignant neoplasm in comparison with post-thyroidectomy histopathological findings among patients who received a thyroidectomy.

## METHODS

This retrospective observational study was conducted in the Pathological Anatomy Laboratory of a private hospital in Joinville, Santa Catarina, Brazil. Cytology results were obtained using FNA, which is considered the gold standard for the diagnosis of malignancies, and were compared to lesion histology. Data were collected from electronic health charts between January 2011 and December 2021.

The inclusion criteria were as follows: patients who underwent FNA and surgical procedures with analysis in the pathological anatomy laboratory. Patients with incomplete medical records were excluded.

The variables analyzed included the age and sex of the patients, size of the nodule, cytological results using the Bethesda system, and histopathological results (benign, papillary carcinoma, follicular carcinoma, or other types). Data collection began after authorization by the Research Ethics Committee, approved by CAAE: 18663219.2.0000.8062; # 3.613.143 of *Hospital Dona Helena* , Joinville, SC, Brazil.

The nodules were classified via ultrasonography according to the ACR Thyroid Imaging Reporting and Data System classification (ACR TI-RADS, 2017) ^( 6 )^ as follows: TR1 (0 or 1 points: benign), TR2 (2 points: not suspicious), TR3 (3 points: mild suspicion), TR4 (4–6 points: moderate suspicion), and TR5 (>6 points: high suspicion).

The FNA results were classified according to the Bethesda System for Reporting Thyroid Cytopathology, ^( 7 )^ as follows: BI, unsatisfactory sample; BII, benign; BIII, atypia of undetermined significance; BIV, suspected follicular neoplasia or follicular neoplasia; BV, suspected malignancy; and BVI, malignant. In addition to FNA interpretation, management may depend on other factors such as clinical, ultrasound, or individual patient preferences. ^( 7 )^ The experience of the clinician performing FNA can impact the cytology results, thereby interfering with the surgical decision.

Concomitant with collection, the data were digitized in an electronic bank. Statistical software (SPSS, version 21.0) was used for data analysis. All variables were analyzed descriptively; means and standard deviations were calculated for continuous variables (numerical). For quantitative variables, absolute and relative frequencies were calculated. To verify the hypothesis of equality between the groups, Student’s *t-* test was used when the distribution was normal, and the non-parametric Mann–Whitney test was used when the data did not pass the Kolmogorov–Smirnov test of normality. The χ ^2^ test or Fisher’s exact test (for groups with frequencies below 5) were calculated to test homogeneity of proportions between groups.

The diagnostic parameters were based on frequency data and concordance between procedures. Nodules classified as Bethesda II were considered benign, whereas nodules classified as Bethesda V and VI were considered malignant. Bethesda classifications I, III, and IV were not included in the statistical calculations for diagnostic evaluation because of indeterminate results. We then calculated the accuracy, sensitivity, specificity, negative predictive value (NPV), positive predictive value (PPV), false-positive rate (FPR), and false-negative rate (FNR). Subsequent analyses were performed using Bethesda III and IV as malignant findings for comparison.

## RESULTS

Three hundred and sixty patients who underwent FNA and post-thyroidectomy histopathological examinations were included. Mean age was 43.6 years and 293 (81.4%) were women. Patient characteristics are summarized in table 1 .

**Table 1:** Characteristics of patients with thyroid nodules who underwent fine-needle aspiration and surgical procedure

	Total (n=360)	Malignant (n=218)	Benign (n=142)	p value
Age, mean (SD)	43.6 (12.0)	43.5 (11.8)	43.8 (12.2)	0.965
<30 years, n (%)	54 (15.0)	31 (14.2)	23 (16.2)	0.608 ^†^
31-40 years, n (%)	91 (25.3)	60 (27.5)	31 (21.8)	0.225 ^†^
41-50 years, n (%)	125 (34.7)	71 (32.6)	54 (38.0)	0.288 ^†^
51-60 years, n (%)	58 (16.1)	39 (17.9)	19 (13.4)	0.255 ^†^
>61 years, n (%)	32 (8.9)	17 (7.8)	15 (10.6)	0.368 ^†^
Sex, n (%)				0.020 ^†^
Male	67 (18.6)	49 (22.5)	18 (12.7)	
Female	293 (81.4)	169 (77.5)	124 (87.3)	
Size of nodule (cm), mean (SD)	2.32 (1.5)	1.9 (1.4)	2.9 (1.4)	<0.001
>1.0, n (%)	299 (83.1)	169 (77.5)	130 (91.5)	0.001 ^†^
>1.5, n (%)	225 (62.5)	109 (50.0)	116 (81.7)	<0.001 ^†^
>2.5, n (%)	139 (38.6)	57 (26.1)	82 (57.7)	<0.001 ^†^
ACR-TIRADS, n (%)				<0.001 ^†^
TR1	8 (2.2)	1 (0.5)	7 (4.9)	0.005 ^‡^
TR2	26 (7.2)	8 (3.7)	18 (12.7)	0.001 ^†^
TR3	59 (16.4)	25 (11.5)	34 (23.9)	0.002 ^†^
TR4	152 (42.2)	104 (47.7)	48 (33.8)	0.009 ^†^
TR5	115 (31.9)	80 (36.7)	35 (24.6)	0.017 ^†^
Bethesda System for Reporting Thyroid Cytopathology, n (%)				<0.001 ^†^
BI	23 (6.4)	11 (5.0)	12 (8.5)	0.197 ^†^
BII	83 (23.1)	10 (4.6)	73 (51.4)	<0.001 ^†^
BIII	50 (13.9)	20 (9.2)	30 (21.1)	0.001 ^†^
BIV	23 (6.4)	7 (3.2)	16 (11.3)	0.002 ^†^
BV	102 (28.3)	92 (42.2)	10 (7.0)	<0.001 ^†^
BVI	79 (21.9)	78 (35.8)	1 (0.7)	<0.001 ^‡^

^†^ χ ^2^ test; ^‡^ Fisher’s exact test.

FNA: fine-needle aspiration.

Of the 360 participants with FNA results, 23 (6.4%) were classified as unsatisfactory (BI), 83 (23.1%) as benign (BII), 50 (13.9%) as atypia of undetermined significance (BIII), 23 (6.4%) as suspected follicular or Hürthle cell neoplasia (BIV), 102 (28.3%) as suspected malignancy (BV) and 79 (21.9%) as malignant (BVI) ( Table 1 ).

After thyroidectomy, histopathological examination classified 142 (39.4%) cases as benign and 218 (60.6%) as malignant. Malignant nodules were more frequently observed in men. Tumor size differed by classification, with malignant tumors being smaller than benign tumors. Among the malignant nodules, 202 (92.6%) were classified as papillary carcinomas, 11 (5.0%) cases as follicular carcinomas, and 5 (2.3%) were classified as other types of carcinomas, such as anaplastic and medullary.

The diagnostic parameters of the Bethesda system on FNA in relation to post-thyroidectomy histopathological evaluation are shown in table 2 . In group I, 11 cases were malignant (47.8%), in group II, 10 cases were malignant (12.0%), in group III, 20 cases were malignant (40.0%), in group IV, 7 cases were malignant (30.4%), in group V, 92 cases were malignant (90.2%), and in group VI, 78 cases were actually malignant (98.7%). Except for Classification I, all Bethesda system categories differed significantly between malignant and benign nodules, as shown in table 1 .

**Table 2:** Comparison of diagnostic parameters from malignant (BV–BVI) *versus* benign (BII) thyroid neoplasms from fine-needle aspiration procedures and histopathology

	FNA malignant (BV–BVI)	FNA benign (BII)
Malignant histopathology	170	10
Benign histopathology	11	73
Parameter	Calculation	Results (%)
Accuracy	243/264	92.04
Sensitivity	170/180	94.44
Specificity	73/84	86.90
Positive predictive value	170/181	93.92
Negative predictive value	73/83	87.95
False positive rate	11/181	6.07
False negative rate	10/83	12.04

n=264. FNA: fine-needle aspiration; BII, BV and BVI: Bethesda System for Reporting Thyroid Cytopathology.

To estimate the diagnostic parameters, Bethesda categories V and VI were considered positive for malignancy, whereas only Bethesda category II was used for negative FNA. The FNA accuracy in our study was 92%, and the sensitivity and specificity found were 94.4% and 86.9%, respectively. The PPV was 93.9% and, 87.9%, with the false positive rate was 6.07%, and false negative 12.04% ( Table 2 ).

When Bethesda categories IV to VI were considered malignant, there was a drop in accuracy of 87.1%, whereas sensitivity and specificity were 94.6% and 73.0%, respectively. Furthermore, the PPV was 86.7% and the false positive rate was 13.2%. However, when considering the Bethesda III to VI categories for malignancy, there was a greater reduction in accuracy to 80.1% compared with sensitivity and specificity of 95.1% and 56.1%, respectively. In this scenario, the PPV was 77.5% and the false positive rate was 22.4%. The results are summarized in tables 3 and 4.

**Table 3:** Comparison of diagnostic parameters from malignant (BIV–BVI) *versus* benign (BII) thyroid neoplasms from fine-needle aspiration procedures and histopathology

	FNA malignant (BIV – BVI)	FNA benign (BII)
Malignant histopathology	177	10
Benign histopathology	27	73
Parameter	Calculation	Results (%)
Accuracy	250/287	87.1
Sensitivity	177/187	94.65
Specificity	73/100	73.0
Positive predictive value	177/204	86.76
Negative predictive value	73/83	87.95
False positive rate	27/204	13.23
False negative rate	10/83	12.04

n=287. FNA: fine-needle aspiration; BII, BIV and BVI: Bethesda System for Reporting Thyroid Cytopathology.

**Table 4:** Comparison of diagnostic parameters from malignant (BIII-BVI) *versus* benign (BII) thyroid neoplasms from fine-needle aspiration procedures and histopathology

	FNA malignant (BIII–BVI)	FNA benign (BII)
Malignant histopathology	197	10
Benign histopathology	57	73
Parameter	Calculation	Results (%)
Accuracy	270/337	80.11
Sensitivity	197/207	95.16
Specificity	73/130	56.15
Positive predictive value	197/254	77.56
Negative predictive value	73/83	87.95
False positive rate	57/254	22.44
False negative rate	10/83	12.04

n=337. FNA: fine-needle aspiration; BII, BIII and BVI: Bethesda System for Reporting Thyroid Cytopathology.

## DISCUSSION

In light of the clinical importance of FNA, the gold standard for the detection of malignant thyroid pathologies and subsequent therapeutic decisions, there is a need to evaluate the accuracy and reliability of this method in comparison with histopathology. Thus, we analyzed the diagnostic parameters of FNA procedures in patients who underwent thyroidectomy and compare these parameters to histopathological results.

Thyroid nodules are a clinical presentation of several diseases and more commonly affect females. ^( 1 )^ Of the 360 patients analyzed, 293 (81.4%) were women and 67 (18.6%) were men with a mean age of 43.5 years. Prior studies had similar patient demographics. Ceratti et al. analyzed data from 94 participants, of whom 90.4% were women with a mean age of 52 years. ^( 4 )^ In a retrospective study conducted from 2010-2019 in Saudi Arabia, 1,543 patients were evaluated of whom 80.5% were women with a mean age of 40.5 years. ^( 8 )^

Although most thyroid nodules are benign, malignancies must always be excluded. ^( 1 )^ In Brazil, the incidence of thyroid carcinoma is 24 cases per 100,000 people. ^( 1 )^ In our study, tumors analyzed through FNA procedures and classified as BV and BVI were more prevalent than in the general population. This can be explained through study methodology, as benign results are rarely referred for thyroidectomy; therefore, they were not included in the present study.

According to the literature, FNA has diagnostic variability owing to sample quality, cases with challenging features, professional experience, and the methodology for classifying lesions. ^( 9 )^ In this context, the accuracy rate of our study was 92.0%, which is similar to that found by Machała et al. ^( 9 )^ and Menegassi et al. ^( 5 )^ which were 89.46% and 89.6%, respectively. These values confirm the high diagnostic performance of FNA as a tool for detecting malignant diseases.

Conversely, we found that the sensitivity and specificity of FNA procedures was 94.44% and 86.90% in our study, respectively. In a study by Alshathry et al. ^( 8 )^ a sensitivity of 69.7% and specificity of 92.9% were observed for detection of malignancy. Nevertheless, it was also observed that sensitivity can vary from 65% to 99%, and specificity has a similar range, from 72% to 100%. ^( 9 )^ Despite these ranges, FNA showed satisfactory results in our study.

Similar studies have been performed in Brazil with limited sample sizes. Torres et al. ^( 10 )^ conducted a single-center retrospective study of 61 patients who underwent cytological analysis and thyroidectomies. The FNA obtained had a sensitivity of 81.2% and specificity of 69.2%, with an accuracy rate of 72.7%. ^( 10 )^ Meanwhile, a cross-sectional study by Canedo et al. ^( 11 )^ analyzed 56 patients undergoing thyroidectomy, with sensitivity of 86.6%, specificity of 88.4%, accuracy rate of 87.5%, with comparable profiles and findings to the present study.

Zocratto ^( 12 )^ discussed reproducibility in the cytological interpretation of FNA through a temporal evaluation and comparison between two examiners with different degrees of experience. A concordance of 89.2% in the intra-examiner analysis was found in contrast to 73% inter-examiner agreement. The examiner’s experience had a significant influence on the proportion of insufficient results and cases compatible with follicular neoplasia. ^( 12 )^

Therefore, surgical indications for thyroid nodules should consider false-negative and insufficient results observed on FNA, which occur even when interpreted by an experienced pathologist, mainly due to the high rates of malignancy observed in these samples. ^( 12 )^

In addition, the technique for performing FNA, whether conventional or ultrasound-guided, can affect the cytology results. Yokozawa ^( 13 )^ found a failure in the diagnosis of malignancy in 15.8% by conventional FNA, in contrast to FNA by ultrasound. The main causes of failure being nodule with difficult palpation in 55.6%), inadequate cytology in 29.2%), and technical difficulty in 15.1%). ^( 13 )^

Regarding the presence of malignancy in each category of FNA results in the groups considered indeterminate, the results were as follows: Bethesda I, 11 malignant cases (47.8% of malignancy, corresponding to 5.0% of all malignant nodules); in Bethesda III, 20 malignant cases (40.0% malignancy, corresponding to 9.2% of all malignant nodules); in Bethesda IV 7 malignant cases (30.4% malignancy, corresponding to 3.2% of the total number of malignant nodules).

Yaprak Bayrak et al. ^( 14 )^ found a malignancy rates of 25% for Bethesda III nodules and 27.6% for Bethesda IV nodules, while in the study by Kraus-Fischer et al. ^( 15 )^ rates of 28% and 36% were detected, respectively. These values demonstrated the low ability of FNA to assess the presence of malignancy when the results fit into an undetermined group. Ultrasound analysis, as well as other complementary examinations, can help in better diagnostic elucidation. Compared with single FNA, sequential biopsy increased sensitivity by 13.8% and specificity by 6.2%, in addition to reducing false negatives and positives by 14.2%, according to a retrospective study by Huang et al. ^( 16 )^ performed with 7,700 patients.

In Bethesda II, 10 cases were malignant (12.0% malignancy, corresponding to 4.6% of all malignant nodules). The occurrence of false negatives in this group was due to the low capacity of FNA to detect changes in less common types of thyroid carcinomas, such as follicular and medullary carcinomas. ^( 9 )^ In these cases, alterations in clinical and complementary examinations must be carefully evaluated. According to the Bethesda System, ^( 7 )^ patients in Group II should not undergo surgery. However, abnormalities that are better evaluated by ultrasound or scintigraphy, such as size, shape, and lymph node involvement, are important in the decision to refer patients for surgery, thereby reducing the chances of new false negatives.

In Bethesda V, 92 cases were malignant (90.2% malignant, corresponding to 42.2% of malignant nodules), whereas in Bethesda VI, 78 cases were indeed malignant (98.7%, corresponding to 35.8% of all malignant nodules). Fine-needle aspiration proved to be an extremely reliable test in these groups. Even so, a high PPV (93.9%) was found when compared to the literature, in which the rates ranged from 75.2% ^( 5 )^ to 90.1%. ^( 9 )^

However, attention should be paid to false positives. We found a false positive rate of 6.0% compared to values below 2% in other studies. ^( 5 , 9 )^ These patients are at risk of overtreatment and overdiagnosis, which must be considered when surgical complications are present in up to 11.3% of thyroidectomies such as hypoparathyroidism (7.5%), paralysis of the recurrent laryngeal nerve (1.9%), in addition to hematomas, and infections. ^( 17 )^ Additionally, complete resection of the thyroid leads to the need for lifelong replacement of thyroid hormones. ^( 17 )^

When Bethesda categories III and IV were added as positive findings for malignancy, accuracy reduced to 80.1% and specificity to 56.1%. Thus, reliability decreases when indeterminate results appear, such as unsatisfactory results or atypia of undetermined significance. On these occasions, the power of FNA to define conduct is low and clinical evaluations associated with other complementary examinations should be performed. ^( 16 )^ In the literature, complementation with molecular tests for classifications III and IV has been suggested, depending on the ultrasonographic characteristics or patient preferences. ^( 18 , 19 )^

The low rate (12%) of false negatives in our study is desirable for diagnostic evaluation of thyroid neoplasms with variable results in the literature, such as Machała et al. ^( 9 )^ (39.7%) or Menegassi et al. ^( 5 )^ (10.5%), which can be attributed to sampling failure and errors in the interpretation of the cytology results. ^( 16 )^

Considering our study evaluated the population of a single private hospital and was conducted retrospectively, there are limitations regarding the applicability of our results in other contexts. Thus, prospective studies are needed to better assess the diagnostic parameters of FNA, mainly focusing on nodules with indeterminate classifications, as this poses a greater clinical challenge.

## CONCLUSION

Fine-needle aspiration is a reliable diagnostic test for distinguishing between benign and malignant thyroid pathologies. For nodules with Bethesda grades I, II, and IV, careful ultrasound evaluation should be performed, which may be associated with a biopsy or other armed workup tests. Considering the clinical importance of fine-needle aspiration, studies reassessing the diagnostic parameters should be performed periodically.
